# Supplementary material for: Utilization of Cottonseed Meal Protein Hydrolysate by Crustaceans: Insights on Growth Performance, Protein Turnover, and Metabolism in Chinese Mitten Crab Eriocheir sinensis
Source: Animals (Basel). 2023 Nov 23;13(23):3631. doi: 10.3390/ani13233631 (PMC10705533; doi:10.3390/ani13233631)

Figure S1.

Igf-1: Full unedited gel for Igf-1 in figure 4.

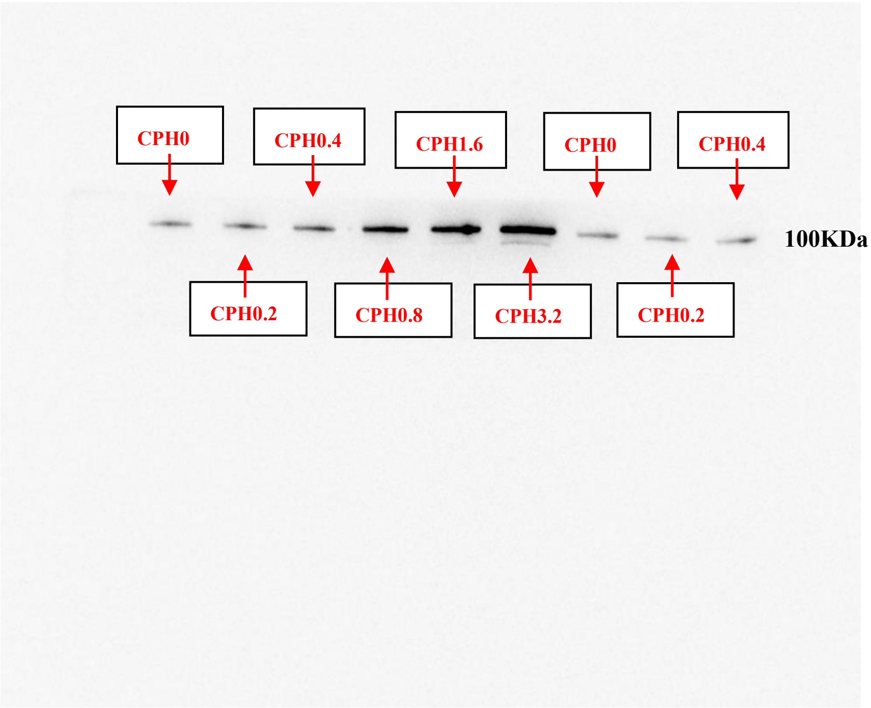

Akt: Full unedited gel for Akt in figure 4.

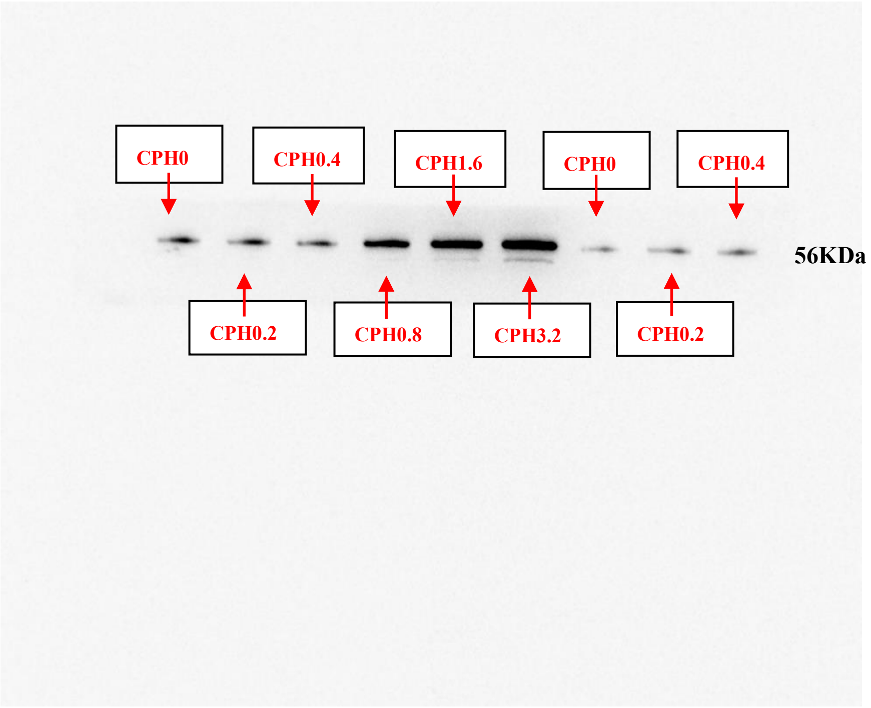

Tor: Full unedited gel for Tor in figure 4.

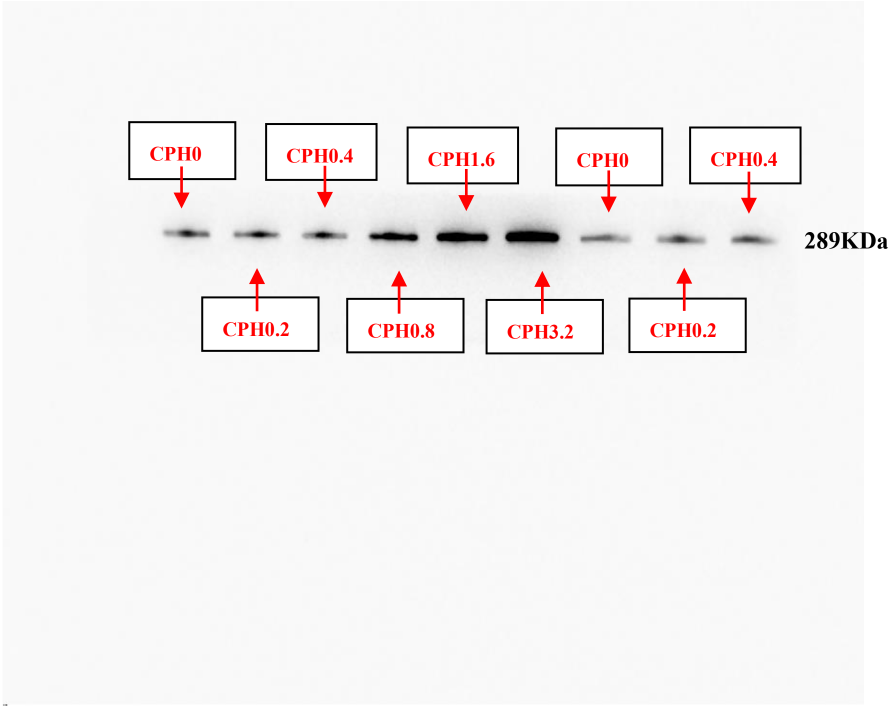

Foxo1: Full unedited gel for Foxo1 in figure 4.

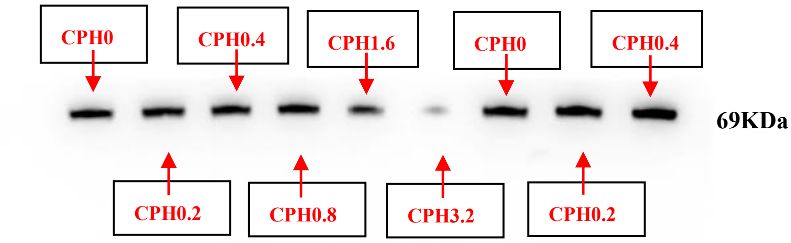

Gapdh: Full unedited gel for Gapdh in figure 4.

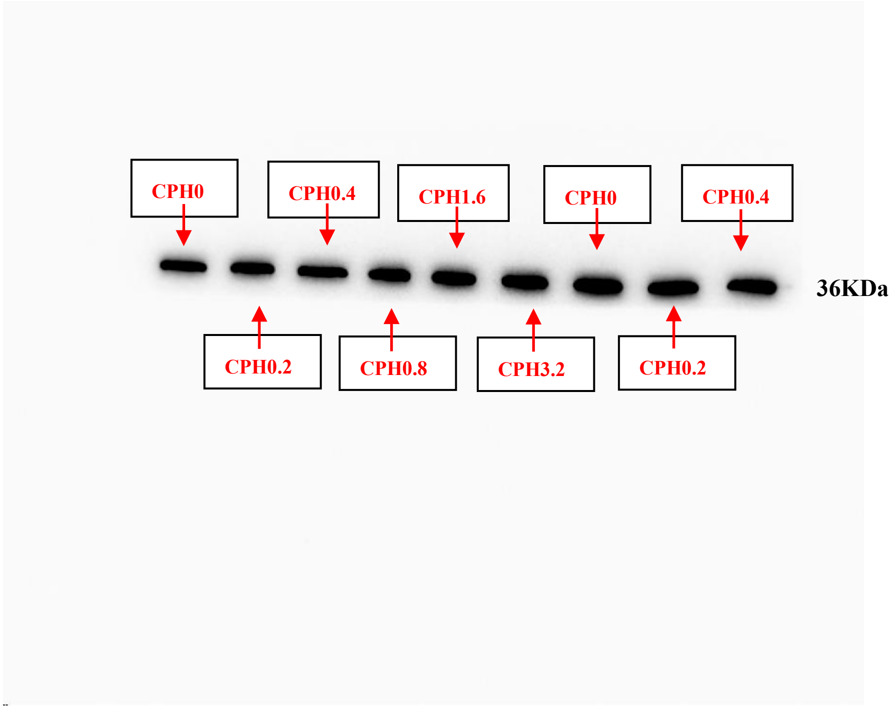

Supplement: Supplementary file 1 [file animals-13-03631-s001.zip › animals-2625057-supplementary.pdf]
